# Transthyretin amyloid cardiomyopathy: from cause to novel treatments

**DOI:** 10.1093/eurheartj/ehaf667

**Published:** 2025-10-01

**Authors:** Marianna Fontana, Alberto Aimo, Michele Emdin, Aldostefano Porcari, Scott D Solomon, Philip N Hawkins, Julian D Gillmore

**Affiliations:** National Amyloidosis Centre, University College London, Royal Free Campus, Rowland Hill Street, London NW3 2PF, UK; Health Science Interdisciplinary Center, Scuola Superiore Sant’Anna, Pisa, Italy; Cardiology Department, Fondazione Toscana Gabriele Monasterio, Pisa, Italy; Health Science Interdisciplinary Center, Scuola Superiore Sant’Anna, Pisa, Italy; Cardiology Department, Fondazione Toscana Gabriele Monasterio, Pisa, Italy; National Amyloidosis Centre, University College London, Royal Free Campus, Rowland Hill Street, London NW3 2PF, UK; Cardiovascular Department, Center for Diagnosis and Treatment of Cardiomyopathies, Azienda Sanitaria Universitaria Giuliano-Isontina (ASUGI), University of Trieste, Trieste, Italy; European Reference Network for Rare, Low Prevalence and Complex Diseases of the Heart (ERN GUARD-Heart), Trieste, Italy; Cardiovascular Division, Brigham and Women's Hospital, Harvard Medical School, Boston, MA, USA; National Amyloidosis Centre, University College London, Royal Free Campus, Rowland Hill Street, London NW3 2PF, UK; National Amyloidosis Centre, University College London, Royal Free Campus, Rowland Hill Street, London NW3 2PF, UK

**Keywords:** ATTR cardiomyopathy, Transthyretin, Cardiac amyloidosis, Clearance, TTR stabilizers, RNA interference, CRISPR–Cas9 gene editing, Antisense oligonucleotides

## Abstract

Transthyretin amyloid cardiomyopathy (ATTR-CM) is a progressive disorder marked by amyloid deposition in the heart, ultimately impairing cardiac function. Recent treatment advances have paralleled an evolving understanding of ATTR-CM pathophysiology. One emerging hypothesis suggests that an imbalance between *in vivo* amyloid production and natural clearance may drive disease progression, a conceptual framework that still requires validation. Transthyretin (TTR) stabilizers, such as tafamidis and acoramidis, mitigate amyloid formation by promoting the native tetrameric conformation of circulating TTR, thereby slowing functional decline and prolonging survival. Similarly, *TTR* gene silencers inhibit hepatic TTR synthesis, and gene-editing therapy with nexiguran ziclumeran offers an additional strategy to reduce amyloid production. However, these approaches do not enhance the body’s limited capacity to clear existing amyloid deposits, underscoring the need for novel agents that accelerate amyloid removal. Promisingly, monoclonal antibodies targeting TTR amyloid are under development, with early clinical trials suggesting that this passive immunotherapy may reverse disease progression and improve heart function. Ultimately, optimal management of ATTR-CM will require further elucidation of the complex interplay between amyloid formation, its structural and functional impacts, its clearance mechanisms, and the potential for myocardial reverse remodelling.

## Graphical Abstract

**Graphical Abstract:**
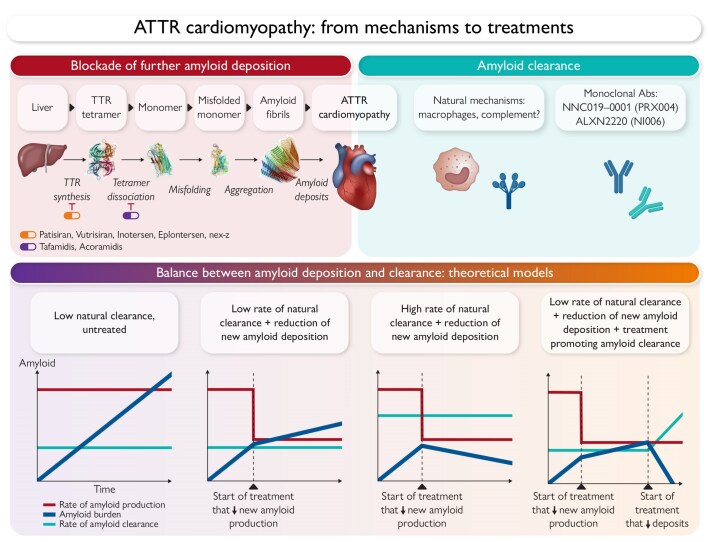
Imbalance between amyloid deposition and clearance as the driver of transthyretin amyloidosis onset and progression: perspectives for treatment. Abs, antibodies; nex-z, nexiguran ziclumeran; TTR, transthyretin.

Systemic amyloidosis represents a diverse group of disorders caused by the deposition of misfolded proteins, arranged as amyloid fibrils within the extracellular space of multiple organs.^1^ To date, more than 40 precursor proteins have been identified that can misfold and self-assemble into insoluble fibrils with a distinctive, highly ordered, abnormal cross beta-sheet structure.^2^ Accurate identification of the amyloid fibril protein is crucial in every case of systemic amyloidosis to guide treatment. Transthyretin amyloidosis (ATTR) involves progressive deposition of fibrillar transthyretin (TTR) protein, whose physiological function includes carrying thyroid hormones and vitamin A. Transthyretin amyloidosis often affects elderly individuals [wild-type ATTR (ATTRwt)], likely because of an age-related decline in the homeostatic mechanisms that regulate protein metabolism. It may also develop because of gene variants reducing the stability of the TTR tetramer [variant ATTR (ATTRv)]. Wild-type ATTR typically presents as a cardiomyopathy (ATTR-CM), whereas ATTRv can manifest with polyneuropathy (PN) and/or cardiac involvement. Prognosis depends on the extent of cardiac amyloidosis coupled with factors such as the patient’s age at symptom onset, the time between initial symptoms and diagnosis, and the specific *TTR* mutation in ATTRv. In the heart, amyloid deposition expands the extracellular matrix, disrupts the myocardial architecture, and impairs systolic and diastolic function.^1^ As myocardial mass increases, the ventricular cavity size diminishes, resulting in a fixed end-diastolic volume. Transthyretin amyloidosis cardiomyopathy (ATTR-CM) progresses slowly and is often well tolerated clinically until significant wall thickening, severe diastolic dysfunction, atrial fibrillation, or conduction abnormalities occur.^1^

Historically, the diagnosis of ATTR-CM required a tissue biopsy. However, the introduction of a non-invasive diagnostic algorithm now enables ∼70% of cases to be diagnosed without the need for histology.^3^ Alongside these advances in diagnostics, treatments that either stabilize the TTR tetramer or reduce its circulating concentration via inhibition of hepatic synthesis have been shown to slow or halt disease progression in ATTRv amyloid PN (ATTRv-PN) and improve survival in ATTR-CM. At the same time, better understanding of the amyloidogenic cascade has driven the development of innovative therapeutic strategies, which, coupled with the ability to diagnose non-invasively, have in turn led to greater disease awareness creating a reinforcing cycle that further incentivizes research, encourages timely intervention, and ultimately leads to improved patient outcomes.

In this review, we explore the latest advances in disease-modifying treatment for ATTR-CM and examine a possible conceptual framework, still requiring validation, considering cardiac disease as the product of an imbalance between amyloid deposition and clearance (*Graphical Abstract*). We conclude by discussing both current and future challenges in ATTR-CM clinical trials.

For this review, relevant studies were searched in PubMed/Medline (updated December 2024) using the following terms: *amyloidosis; cardiac amyloidosis; transthyretin; ATTR; cardiomyopathy; stabilizer; siRNA; ASO; silencer; gene editing* as well as the drug names. We also searched for press releases about each drug. Given the design of this work as a narrative review, no formal criteria for study selection or appraisal were enforced.

## Amyloidosis as the product of an imbalance between production and clearance

Serum amyloid P (SAP) component scintigraphy, a quantitative diagnostic and monitoring tool for visceral amyloid deposits,^4^ and cardiac magnetic resonance imaging, a means of tracking cardiac amyloid burden via serial measurement of extracellular volume,^5^ have demonstrated that amyloid deposits exist in a state of dynamic equilibrium dependent upon the rates of ongoing amyloid formation and natural amyloid clearance.^5,6^ Because amyloid clearance *in vivo* is consistently slow, amyloid formation in untreated patients likely outpaces its clearance, resulting in progressive accumulation and a decline in organ function.

Therapies that suppress the circulating concentration of the respective amyloid fibril precursor protein reduce the substrate available required for new amyloid formation.^1^ In the case of ATTR amyloidosis, ongoing amyloid formation can also be inhibited by drugs that stabilize the normal soluble TTR tetramer, thereby reducing the availability of misfolded TTR species that serve as the substrate for ATTR amyloid fibril formation.^7^ There are currently no biological assays that specifically measure the rate of amyloid formation or the rate of amyloid clearance *in vivo*.^8^ However, serial quantitative imaging in patients with systemic light-chain (AL) amyloidosis who have achieved a complete haematological response^9^ as well as in patients with hereditary fibrinogen A α-chain amyloidosis who have undergone liver transplantation such that they have no ongoing amyloid formation^10^ has demonstrated that, although the rate of amyloid clearance is consistently slow, it varies substantially between patients and even between different organs in the same patient. For example, amyloid clearance from the liver and spleen typically occurs more rapidly than it does from the heart and tongue.^9^

It is perhaps surprising that the clearance of amyloid, with its acquired highly abnormal structure, occurs so slowly; the reasons for this and mechanisms of clearance remain uncertain. Histological evaluation of patients’ biopsies shows that amyloid deposits usually, but not always, elicit a very limited inflammatory response.^11^ In contrast, studies in mice with experimentally induced AA amyloidosis^12^ and in humans with various types of systemic amyloidosis^13,14^ have shown that clearance of visceral amyloid can be markedly accelerated following administration of antibodies targeting specifically the amyloid deposits. Based on evidence from the murine model, accelerated antibody-mediated amyloid clearance involves activation of the classical complement pathway, recruitment of macrophages, and formation of multinucleated giant cells that engulf surrounding amyloid. This process is abrogated when there is a deficiency in complement and/or macrophages.^12^ These experimental findings were supported by human evidence, as complement activation was observed in patients with systemic amyloidosis who experienced rapid visceral amyloid clearance following administration of the anti-SAP antibody dezamizumab.^15^ The fact that age at disease onset in carriers of the pathogenic p.Val50Met TTR variant is influenced by functional variants of C1q lends further support to the potential role of the complement pathway in amyloid pathogenesis.^16^

Modest ATTR amyloid deposition is common in the elderly,^17^ yet only a small proportion develop sufficient deposition to cause clinically significant cardiomyopathy.^18^ Amyloid accumulation can result from increased production, reduced clearance, or both. Potential mechanisms include age-related changes that reduce TTR tetramer stability or alter its tissue interactions, leading to increased deposition, and/or reduced amyloid clearance. Further research is needed to clarify these mechanisms and their relative contributions in different patient subsets.

## Therapies reducing amyloid accumulation

**Figure 1:**
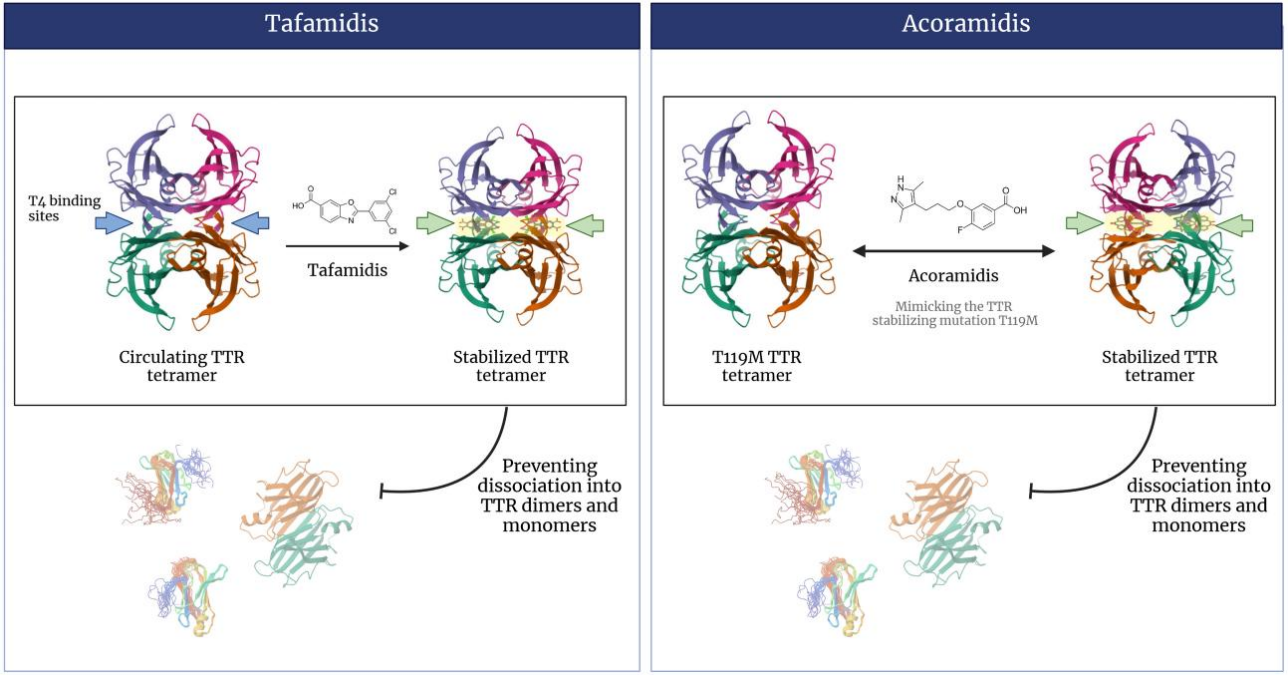
Transthyretin stabilizers: mechanism of action. Transthyretin stabilizers (tafamidis and acoramidis) bind to transthyretin tetramers at the thyroxin-binding sites and slow dissociation of the transthyretin tetramer into its constituent monomers, the rate-limiting step in amyloidogenesis

**Figure 2:**
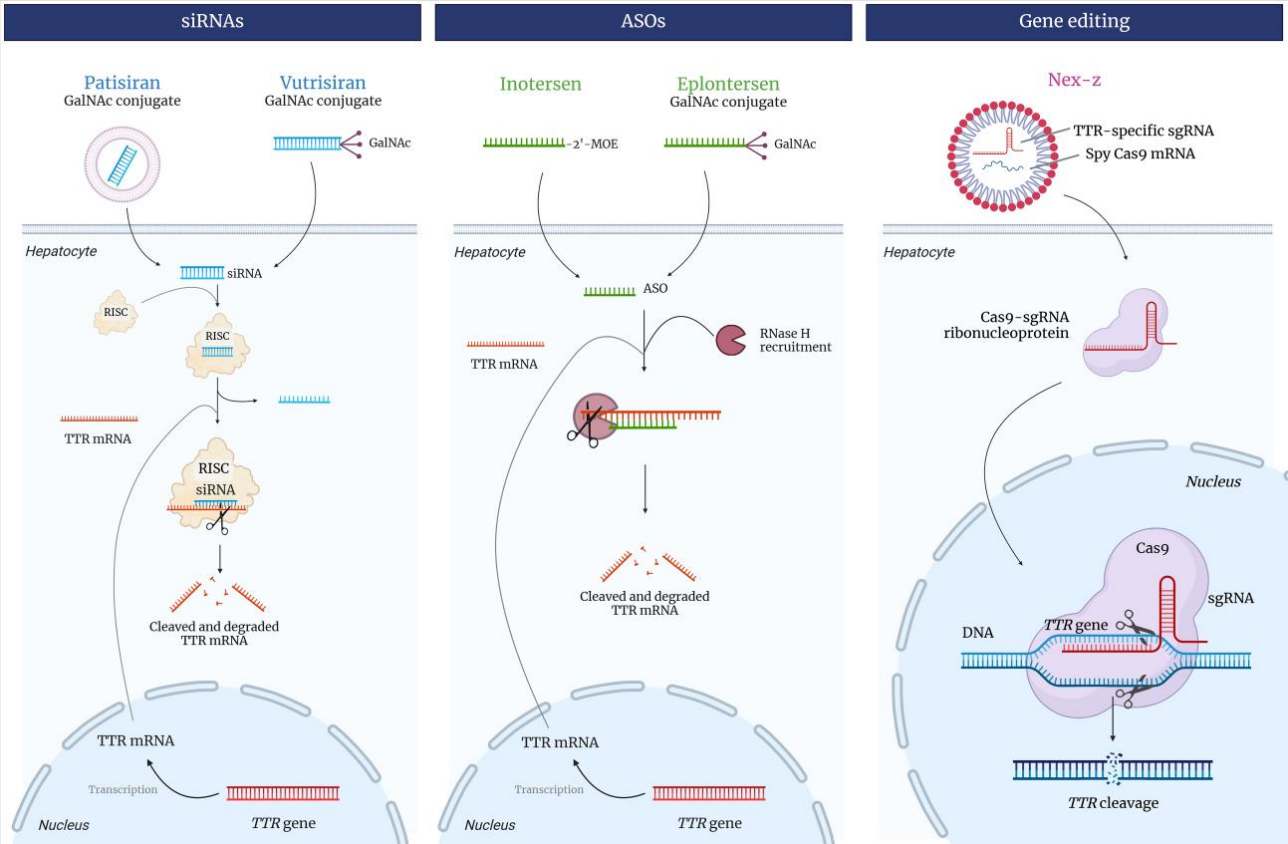
Inhibitors of transthyretin synthesis: mechanisms of action. Small interfering RNAs and antisense oligonucleotide promote the degradation of the transthyretin messenger RNA through different molecular mechanisms, schematically displayed in the figure. The final effect is a blockade of transthyretin synthesis. Cas, caspase; GalNAc, *N*-acetylgalactosamine; RISC, RNA-induced silencing complex; sgRNA, single-guide RNA

The primary goal of all approved therapies for amyloidosis is preventing further amyloid deposition in tissues to slow or halt disease progression. In ATTR amyloidosis, this can be achieved by stabilizing TTR (*Figure 1*) or by reducing its synthesis (*Figure 2*).

### Transthyretin stabilizers

Tafamidis is a small-molecule, oral TTR stabilizer. It binds to the T4-binding sites on the TTR tetramer, reducing its dissociation into monomeric TTR, the substrate for ongoing amyloid formation.^19^ In a Phase 3 trial (ATTR-ACT), tafamidis treatment in patients with ATTRwt- and ATTRv-CM-related heart failure (HF) reduced all-cause mortality and cardiovascular hospitalization by around 30% compared with placebo, although the survival curves of patients treated with tafamidis or on placebo started to diverge only after 18 months. Notably, patients in New York Heart Association (NYHA) Class IV were excluded from this study, and patients in NYHA Class III receiving tafamidis displayed a higher risk of cardiovascular hospitalization. Compared with placebo, the drug slowed the decline in both functional capacity and quality of life and proved safe and well tolerated.^20^

Tafamidis became the first disease-modifying agent to receive approval for the treatment of both ATTRwt- and ATTRv-CM.^21^ The long-term extension (LTE) study of ATTR-ACT showed a 36% lower mortality in patients with NYHA Class III at baseline who received continuous tafamidis in ATTR-ACT and the LTE study when compared with those who received placebo in ATTR-ACT and tafamidis in the LTE study, suggesting a prognostic benefit even in patients with NYHA Class III symptoms.^22^ Since no data on cardiovascular hospitalizations were provided, it is impossible to check whether the increase in cardiovascular hospitalizations was confirmed or to speculate on its possible causes. Real-world studies have reinforced the clinical benefits of tafamidis in patients with ATTR-CM. Data from the Transthyretin Amyloidosis Outcomes Survey revealed that tafamidis-treated patients experienced significantly higher survival rates at 30 and 42 months compared with untreated patients, supporting its efficacy outside of controlled clinical trials.^23^ In addition, an international cohort study focusing on octogenarians with ATTRwt-CM demonstrated that tafamidis treatment improves survival even in older patients, although its benefit may be diminished in those aged 90 years or above or with advanced disease stages.^24^ Complementing these findings, a multicenter study from the USA reported encouraging long-term outcomes, with a 65-month survival probability exceeding 50% in a contemporary cohort of tafamidis-treated patients.^25^ Additionally, multiple real-world studies have confirmed the positive effects of tafamidis on exercise capacity and even reported positive effects on cardiac remodelling. A recent meta-analysis pooling all the available evidence found better global longitudinal strain on echocardiography, cardiac uptake of a bone tracer, and extracellular volume on magnetic resonance imaging in patients on tafamidis.^26^

Acoramidis is a novel TTR stabilizer designed to mimic the effect of the Thr119Met TTR variant and achieves near-complete TTR stabilization.^27^ In the Phase 3 ATTRibute-CM trial, acoramidis demonstrated significant benefits, including an improvement in the composite hierarchical primary endpoint encompassing all-cause mortality, cardiovascular hospitalization, change from baseline in N-terminal pro-B-type natriuretic peptide (NT-proBNP), and change from baseline in 6-min walk distance (6MWD). Subgroup analyses did not show an increase in cardiovascular hospitalizations in patients with NYHA Class III who were on acoramidis. Furthermore, patients treated with acoramidis showed higher circulating TTR levels compared with those treated with placebo and tafamidis, highlighting the role of TTR concentration as a potential circulating biomarker of TTR stabilization and therapeutic efficacy.^28^ This increase in serum TTR was sustained over 30 months and further enhanced in patients who transitioned from placebo plus tafamidis to acoramidis in the open-label extension study.^29^ To our knowledge, no study has yet explored the association between the extent of the increase in circulating TTR and either better clinical status or improved cardiovascular outcomes. Consequently, it remains unclear whether a specific increase in TTR levels can be deemed clinically meaningful and help differentiate responders from non-responders and stratify patient risk.

### Reducing transthyretin synthesis

Circulating TTR plays a critical role in disease development. It is encoded by a single gene and synthesized almost exclusively by the liver.^8^ Because its physiological functions overlap with those of other proteins, reducing serum TTR appears to be safe, provided that vitamin A supplementation is administered. Importantly, inhibiting TTR production in the liver does not affect its synthesis in the retina or central nervous system.^8^ Although the potential long-term adverse effects of TTR suppression cannot be entirely ruled out, ATTR remains an ideal model for strategies aimed at blocking the synthesis of a precursor protein.

Small interfering RNAs (siRNA) are small non-coding double-stranded RNA molecules whose mechanism of action is via post-transcriptional gene silencing through binding of complementary mRNA molecules that induce degradation.^30^ The net effect is a significant reduction in the production of TTR protein by hepatocytes and decreased circulating TTR concentration.^31^ Patisiran is a siRNA encapsulated in lipid nanoparticles that inhibits TTR (either wt or variant) production in hepatocytes.^31^ The APOLLO-B trial randomized patients with ATTR-CM to patisiran or placebo. Over 12 months, patients on patisiran displayed a reduction in decline from baseline in 6MWD (*P* = .02) and in Kansas City Cardiomyopathy Questionnaire overall summary (KCCQ-OS) score (*P* = .04) compared with those on placebo; however, the clinical relevance of these statistically significant but relatively small differences was not clear.^32^ As a result, patisiran was denied approval for treatment of ATTR-CM by the US Food and Drug Administration (FDA).^33^ Vutrisiran is a TTR-specific siRNA with enhanced stabilization chemistry that is administered subcutaneously every 12 weeks. Following promising results with vutrisiran in patients with ATTRv-PN and cardiac involvement in the HELIOS-A trial^34^ (see below), vutrisiran was investigated in the Phase 3 HELIOS-B trial. This trial included 655 participants randomized to receive either vutrisiran or a placebo over 36 months. Vutrisiran significantly reduced the risk of all-cause mortality and recurrent cardiovascular events compared with placebo. It also showed benefits in preserving functional capacity and quality of life, as assessed by 6MWD and KCCQ-OS score. The treatment was effective in both the overall population and a subgroup not receiving concomitant tafamidis at baseline, as well as in most subgroups. Adverse event rates were comparable between the drug and placebo groups in the trial.^35^ The FDA has recently approved vutrisiran for the treatment of ATTR-CM.^36^ Nucresiran (formerly ALN-TTRsc04) is another siRNA being investigated. In a Phase 1 study, a single dose of ≥300 mg achieved rapid TTR knockdown with reductions sustained through at least Day 180 and above 70% at Day 360 for the 300 mg dose. The treatment showed low inter-patient variability and was well tolerated (Murad A., American Heart Association Scientific Sessions 2024, unpublished data).

Antisense oligonucleotides (ASOs) consist of short nucleotide sequences that bind to complementary mRNAs, promoting their degradation and thereby lowering circulating TTR concentration.^31^ In the Phase 3 NEURO-TTR trial, weekly subcutaneous injections of the ASO inotersen for 15 months slowed the neurological and quality of life decline in patients with ATTRv-PN.^37^ Variant ATTR-CM was present in 63% of the study population. No significant changes in left ventricular systolic or diastolic function were observed in the trial.^37^ Conversely, in a single-centre study of patients with both ATTRwt and ATTRv-CM followed for 24 months, inotersen treatment reduced mean left ventricular mass by 8% [as measured by cardiac magnetic resonance (CMR)] and improved exercise capacity as evidenced by a 20-m increase in 6MWD from baseline.^38^ Adverse events documented in the NEURO-TTR trial included glomerulonephritis and severe thrombocytopenia in ∼3% of patients,^37^ which were sufficient to prevent further research on inotersen as a possible therapy for ATTR-CM. The ongoing Phase 3 CARDIO-TTRansform trial (NCT04136171) is investigating eplontersen, an ASO conjugated to a different structure, which can be administered by monthly subcutaneous injection. This trial is the largest so far in ATTR-CM, with over 1400 patients enrolled. Key features of this trial also include large CMR and scintigraphy substudies. The results are expected in 2026.^39^

An innovative strategy for inhibiting TTR synthesis involves *in vivo* ‘editing’ of the *TTR* gene using the clustered regularly interspaced short palindromic repeats and associated Cas9 endonuclease (CRISPR–Cas9) system. Nexiguran ziclumeran (nex-z), formerly known as NTLA-2001, was designed as a one-time treatment to achieve durable TTR reduction by directly targeting the *TTR* gene in hepatocytes. Once inside the hepatocyte nucleus, it activates endogenous DNA repair mechanisms via non-homologous end joining, introducing indels into the *TTR* gene. These indels induce frameshift mutations that inhibit the production of functional TTR protein, ultimately resulting in a profound and sustained decrease in serum TTR concentration.^40^ In a Phase 1, open-label trial, 36 patients with ATTR-CM received a single intravenous infusion of nex-z.^41^ Rapid, profound, and sustained reductions in serum TTR levels were observed. At 28 days post-infusion, the mean reduction in serum TTR was 89%, which remained consistent at 90% at the 12-month follow-up. These reductions were observed across all patients, regardless of disease severity or genotype, and were sustained for up to 24 months in a subset of patients. The safety profile of nex-z was favourable, with no unexpected adverse events or treatment discontinuations and no clinical evidence of off target editing. Infusion-related reactions were manageable and transient, and liver enzyme elevations were mild and self-limiting. In terms of clinical outcomes, a preliminary analysis over 12 months demonstrated stable disease in most patients across the evaluation period (as opposed to an expected decline). N-terminal pro-B-type natriuretic peptide and cardiac troponin T levels, which typically worsen in untreated ATTR-CM, remained stable. Similarly, functional assessments showed a median increase in 6MWD of 5 m in the study population with 92% achieving a stable or improved NYHA functional class across the study period accompanied by stability of the main parameters evaluated by cardiopulmonary exercise testing. Imaging assessments, including CMR, showed stability in cardiac structure and extracellular volume corroborating the clinical findings.^41^ Longer-term follow-up is essential to assess the durability of TTR suppression, the long-term safety of CRISPR-based gene editing, and its impact on clinical outcomes. The ongoing Phase 3 MAGNITUDE trial (NCT06128629) will provide some of these data.

## Circulating transthyretin: the lower, the better?

A relationship between greater reduction in circulating TTR and better clinical outcomes could be hypothesized based on data from other forms of amyloidosis. For example, there is a clear relationship between the magnitude of serum amyloid A (SAA) knockdown and risk of death in AA amyloidosis.^6^ The relative risk of death is nearly four times higher among patients with a median SAA concentration between 4 and 9 mg/L compared with those with a median SAA of <4 mg/L (i.e. a normal SAA concentration), underscoring the importance of normaling the production of the amyloid fibril precursor protein. Persistence of excessive inflammation as measured by a median SAA concentration of >155 mg/L was associated with a near 18-fold risk of death.^6^ Similarly, in AL amyloidosis, outcomes are significantly better in patients who achieve a haematological complete response with chemotherapy (equivalent to 100% knockdown of the circulating amyloidogenic protein) than in patients who achieve a very good partial response (equivalent to at least 90% knockdown). In turn, patients who achieve a very good partial response do better than those who achieve a partial response, equivalent to 50–90% knockdown, who do better than those who achieve <50% knockdown. Most notably, in patients with AL amyloidosis, those who achieve a complete haematological response (characterized by undetectable levels of the precursor protein) demonstrate the best survival rates compared with those with lesser degrees of reduction in circulating fibril precursor protein concentration following chemotherapy.^42^ An association between the magnitude of reduction in circulating TTR achieved with patisiran and change in neuropathy score as a measure of clinical outcome has been demonstrated in patients with ATTRv-PN.^43^ On the other hand, an association between the magnitude of TTR reduction and the improvement in cardiac parameters or cardiac outcome has never been reported in patients with ATTR-CM.

**Figure 3:**
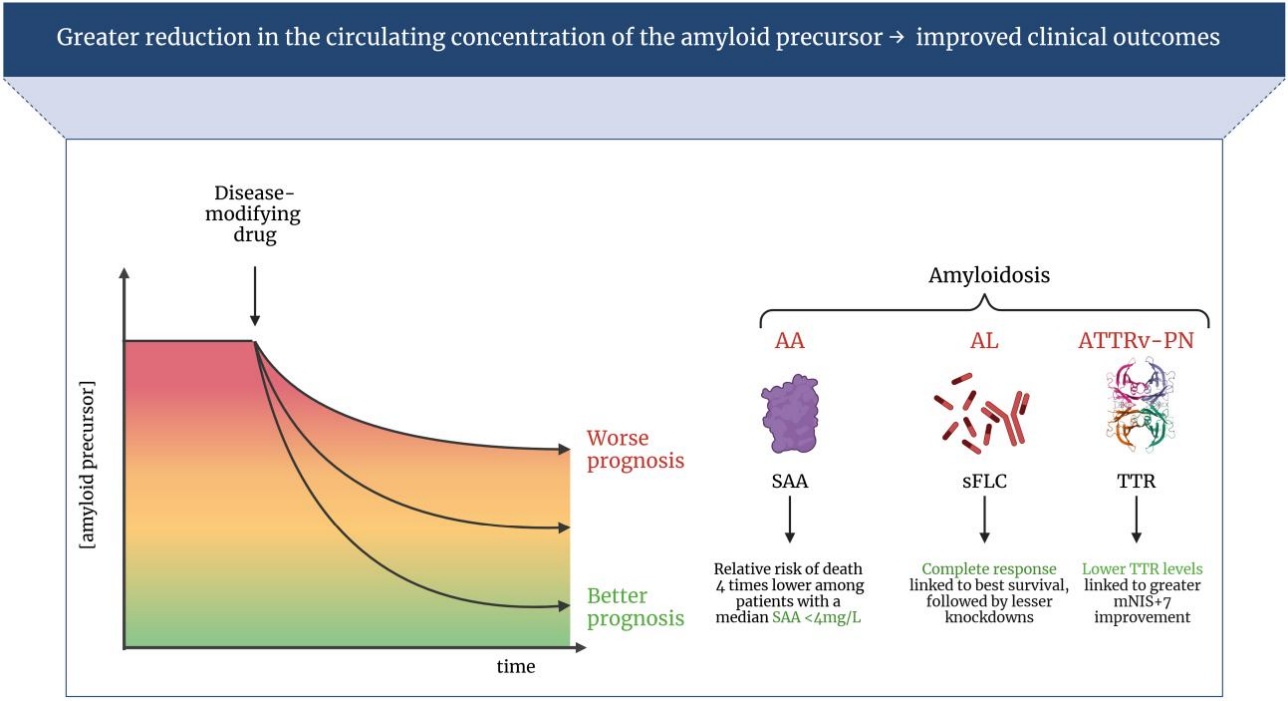
Amyloid precursor protein and outcomes. Data about amyloid A amyloidosis are taken from Lachmann *et al*.,^6^ those about light-chain amyloidosis from Palladini *et al*.,^30^ and those about variant transthyretin amyloidosis with polyneuropathy from the APOLLO-A trial.^43^ SAA, serum amyloid A; sFLC, serum free light-chain

To summarize, we propose a conceptual framework, informed by experience with AL and AA amyloidosis as well as preliminary data in ATTRv-PN amyloidosis, suggesting that a greater magnitude and consistency of reduction in serum TTR concentration will lead to better clinical outcomes in patients with ATTR-CM (*Figure 3*).

## Antibody-mediated amyloid removal: from anecdotal experience to a viable therapeutic strategy

The natural mechanisms of amyloid removal described in the previous section are intriguing, but their relative contribution in the heart and for preventing build-up of ATTR amyloid is unclear. Strategies that enhance amyloid removal by eliciting a targeted immune response hold considerable promise.

ALXN2220 (formerly NI006) is a human-derived monoclonal IgG1 antibody that binds to the linear epitope WEPFA, which is only accessible on misfolded TTR and ATTR deposits, triggering phagocytosis of ATTR aggregates by human macrophages thereby accelerating fibril removal.^44^ In the first-in-human, randomized, double-blind, placebo-controlled, Phase 1 trial, patients with ATTR cardiomyopathy and chronic HF received intravenous ALXN2220/NI006 infusions or placebo every 4 weeks over a 4-month period, followed by an 8-month open-label extension phase.^45^ Participants were assigned to increasing dose levels ranging from 0.3 to 60 mg/kg. All enrolled patients had a confirmed diagnosis of ATTR-CM, a minimum left ventricular wall thickness of 14 mm, preserved ejection fraction of at least 40%, and a range of mild-to-moderate HF symptoms. The majority were receiving concomitant treatment with tafamidis, but no other ATTR-specific therapies were permitted. Safety and tolerability served as the primary objectives, with dose escalation contingent on the absence of clinically significant adverse events. Among 40 enrolled patients, there were no clearly drug-related serious adverse events, and no antidrug antibodies were detected. Most adverse events were mild or moderate and considered typical of the disease in this patient population. A few patients showed mild cytokine release syndrome or transient musculoskeletal symptoms, such as arthralgias, which generally resolved with standard symptomatic management. Some patients had transient asymptomatic thrombocytopenia, one case leading to trial discontinuation. Pharmacokinetic assessments indicated that ALXN2220/NI006 behaved like a typical human IgG antibody with a half-life of 2–3 weeks. Imaging studies revealed dose-dependent reductions in cardiac amyloid load over time. Patients receiving at least 10 mg/kg of ALXN2220/NI006 every 4 weeks for up to 1 year showed reduced extracellular volume, suggesting a decreased amyloid burden. Conversely, those receiving placebo showed no such improvements until they too switched to active treatment in the extension phase. Additional analyses suggested that high doses of ALXN2220/NI006 might be associated with improvements in cardiac biomarkers such as NT-proBNP and troponin T, which are linked to disease severity and prognosis in ATTR-CM.^45^ Although these findings were based on a small sample and must be interpreted with caution, they are consistent with an accelerated removal of pathogenic amyloid deposits leading to potential improvements in cardiac function. ALXN2220 is being investigated in a Phase 3 trial (NCT06183931).

NNC6019-0001 (former known as PRX004) is a humanized monoclonal antibody that targets misfolded monomeric and aggregated forms of TTR, promoting antibody-mediated phagocytosis and amyloid clearance. A Phase 2 trial initiated in 2022 aims to evaluate the efficacy and safety of NNC6019-0001 in 99 patients with ATTR-CM. Participants are receiving either NNC6019-0001 at 30 or 100 mg/kg, or placebo on top of standard-of-care treatment. The study will examine changes in the 6MWD and NT-proBNP levels at Week 52 along with changes in structural and functional cardiac parameters, the incidence of cardiovascular hospitalizations and urgent HF visits, and patient-reported quality of life.^46^

## Future perspectives

**Figure 4:**
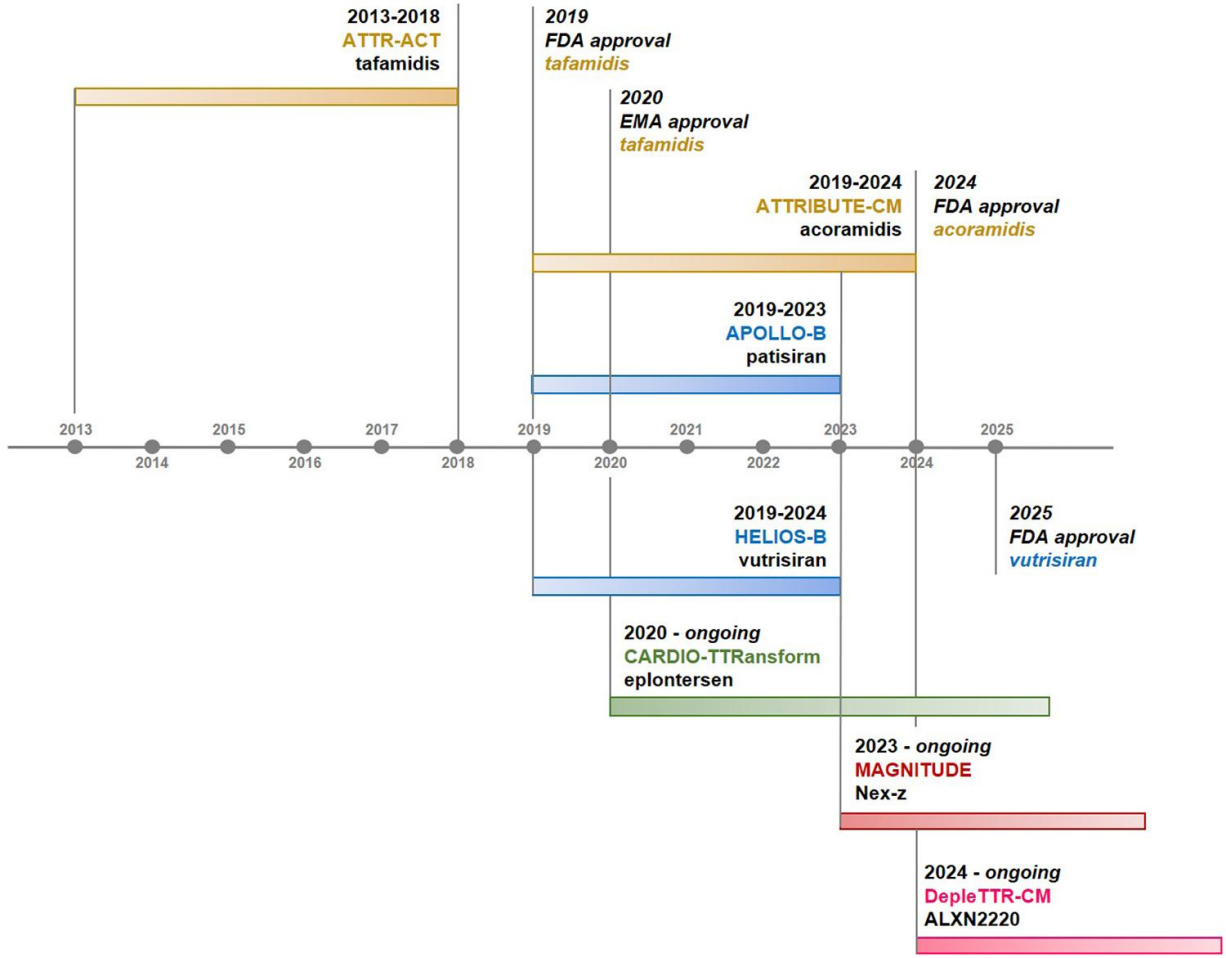
Evolving landscape of treatment for transthyretin amyloid cardiomyopathy. Completed and ongoing Phase 3 trials are reported together with the years of approval by the Food and Drug Administration or European Medicines Agency

Several therapeutic options for ATTR-CM are currently under investigation (*Table 1* and *Figure 4*). Pharmacologic TTR stabilizers and silencers have already shown that slowing amyloid deposition can prolong survival and enhance quality of life. However, critical questions remain—namely, which therapy offers the greatest benefit, how best to tailor treatment to each patient, and what criteria should define a clinically meaningful response. The absence of direct, head-to-head comparisons only underscores the importance of specialized, multidisciplinary care that tailors therapy to individual patient needs.

**Table 1:** Approved or investigational therapies for transthyretin amyloidosis

	ATTRv-PN: clinical use	ATTR-CM			
		Phase 1	Phase 2	Phase 3	Clinical use
Inhibitors of ATTR accumulation					
*TTR stabilizers*					
Tafamidis	Approved			Completed	Approved (FDA, EMA)
Acoramidis				Completed	Approved (FDA, EMA)
*Blockers of TTR synthesis*					
Patisiran	Approved			Completed	Not approved
Vutrisiran	Approved			Completed	Approved (FDA)
Nucresiran		Completed			
Inotersen	Approved				
Eplontersen	Approved			Ongoing	
Nexiguran ziclumeran		Ongoing		Ongoing	
Promoters of ATTR amyloid degradation					
*Monoclonal antibodies*					
PRX004			Ongoing		
ALXN2220 (NI006)				Ongoing	

CM, cardiomyopathy; EMA, *European Medicines Agency;* FDA, Food and Drugs Administration; PN, polyneuropathy.

An emerging strategy to maximize efficacy involves combination therapies that address multiple disease mechanisms. For example, agents that reduce amyloid formation via TTR knockdown can be paired with stabilizers targeting the remaining circulating TTR to curb new deposition. Yet, the notion that fully halting amyloid formation would shift the balance towards net amyloid clearance remains unproven, as no current intervention completely blocks deposition. Forthcoming clinical trials, particularly those evaluating next-generation agents such as nucresiran and gene-editing approaches, will help clarify whether deeper TTR suppression leads to larger reductions in amyloid load and more significant clinical gains. If gene editing is shown to be both safe and durable as a one-time intervention, it could redefine the treatment landscape, contingent on robust long-term outcomes and feasibility analyses. At the same time, insights into intrinsic amyloid clearance pathways might unlock new therapeutic avenues, whether by harnessing or augmenting these endogenous processes. Whether these or other modalities can achieve rapid and complete amyloid clearance (a result that could simplify or shorten the duration of therapy) remains to be seen.

Pursuing such innovative treatments also raises concerns regarding cost, access, and equity. High-complexity interventions, like gene editing in conjunction with amyloid clearance, could carry substantial financial implications, making careful patient selection essential to ensure both cost-effectiveness and avoidance of futile therapy. Younger individuals, those with earlier-stage disease, or those poised to benefit from sustained improvements in cardiac function may emerge as prime candidates. Striking a balance between therapeutic ambition and fiscal reality is crucial to ensure equitable access for all who stand to benefit.

Supportive HF therapies are likewise central to symptom management in ATTR-CM. Along with disease-specific treatments, established HF regimens (such as renin–angiotensin–aldosterone system blockers, beta-blockers, and mineralocorticoid receptor antagonists) continue to play a key role in symptom control and improving outcomes.^47^ Notably, sodium–glucose cotransporter-2 inhibitors have shown promise in reducing hospitalizations and mortality in HF,^48^ highlighting the value of a comprehensive strategy that merges targeted and supportive interventions.

In summary, while therapeutic innovations have markedly broadened the range of options for patients with ATTR-CM, pressing questions about optimal treatment, patient selection, and response metrics highlight the need for ongoing research and direct clinical comparisons. Until those data become available, individualized, interdisciplinary management remains paramount for delivering effective, patient-centered care.

## Declarations

### Data Availability

No data were generated or analysed for or in support of this paper.
